# Evaluation of extracorporeal membrane oxygenation in children with acute hypoxemic respiratory failure and hemodynamic instability in China: a 7-year single-center retrospective study

**DOI:** 10.3389/fmed.2026.1867681

**Published:** 2026-06-24

**Authors:** Jiayun Ying, Xiaodi Cai, Gangfeng Yan, Ye Cheng, Weiming Chen, Guoping Lu

**Affiliations:** 1 Department of Critical Care Medicine, Children’s Hospital of Fudan University, National Children’s Medical Center, Shanghai, China; 2 Shanghai Institute of Infectious Disease and Biosecurity, Fudan University, Shanghai, China; 3 National Health Commission Key Laboratory of Neonatal Diseases, Fudan University, Shanghai, China

**Keywords:** acute hypoxemic respiratory failure, extracorporeal membrane oxygenation, hemodynamic instability, P/F ratio, pediatric intensive care unit

## Abstract

**Objectives:**

To characterize the clinical features, laboratory parameters, and outcomes of pediatric patients with acute hypoxemic respiratory failure (AHRF) and concurrent hemodynamic instability requiring extracorporeal membrane oxygenation (ECMO), and to identify risk factors associated with mortality.

**Methods:**

This retrospective study was conducted in the Pediatric Intensive Care Unit (PICU) of the Children’s Hospital of Fudan University, China. We enrolled patients aged 28 days–18 years with AHRF and hemodynamic instability despite mechanical ventilation who received ECMO support between January 2015 and December 2021. The primary outcome was 28-day in-hospital mortality. Data including demographics, comorbidities, laboratory findings, ventilator settings, and rescue therapies were analyzed. Multivariable logistic regression analysis was used to identify independent risk factors for survival. Receiver operating characteristic (ROC) curves and Kaplan-Meier survival curves were subsequently constructed to evaluate the predictive performance and compare cumulative survival rates, respectively.

**Results:**

Out of 57 eligible patients, 52 (91.2%) were included in the final analysis. The overall mortality rate was 53.8%. Successful weaning, achieved in 34 patients, correlated strongly with improved survival (*p* < 0.001). Before ECMO initiation, survivors had significantly higher PaO_2_ (median: 64.4 vs. 60.5 mmHg, *p* = 0.011) and P/F ratio (75.9 vs. 61.8, *p* = 0.007) than non-survivors. The P/F ratio emerged as a significant predictor of survival on ROC analysis, yielding an AUC of 0.766 (95% CI: 0.629 and an optimal cut-off 77.9.

**Conclusion:**

For pediatric patients with severe AHRF and hemodynamic compromise placed on VA-ECMO, lower pre-ECMO PaO_2_ and P/F ratio were associated with poor prognosis.

## Introduction

Acute hypoxemic respiratory failure (AHRF), encompassing acute respiratory distress syndrome (ARDS), accounts for a leading proportion of pediatric intensive care unit (PICU) admissions (1, 2). While PICU mortality rates for AHRF have declined over the past four decades, respiratory failure remains a primary contributor to death in children under 5 years of age globally (3). Unlike adults, pediatric patients possess distinct physiological profiles; those with severe AHRF frequently present with concomitant hemodynamic instability. Despite advances in care, mortality in this subgroup remains high, necessitating further optimization of rescue strategies.

Extracorporeal membrane oxygenation (ECMO) provides temporary mechanical support for refractory cardiac or pulmonary failure. Since its inception in the 1960s (4), ECMO technology has advanced significantly. While adult trials have demonstrated the efficacy of veno-venous (VV) ECMO for respiratory failure (5), the pediatric literature still lacks large-scale randomized controlled trials. Nevertheless, observational data supported ECMO as a life-saving intervention, with current registries tracking survival rates following pediatric respiratory ECMO between 56% and 72% (6, 7).

Recent data from China suggest survival and safety advantages for VV-ECMO over veno-arterial (VA) ECMO in pediatric ARDS (8). However, children presenting with both AHRF and hemodynamic instability represent a distinct clinical challenge where VV-ECMO alone may not provide sufficient cardiopulmonary support. The literature offered scarce data regarding the optimal application of VA-ECMO in this specific population. Consequently, this study aimed to evaluate the clinical characteristics, laboratory parameters, and outcomes of pediatric patients with AHRF and hemodynamic compromise undergoing VA-ECMO support, while identifying specific risk factors associated with mortality. A prior preprint archives the preliminary findings of this work (9).

## Materials and methods

### Study design

This retrospective study was conducted in the PICU of the Childrensupport, while Fudan University, China. We aimed to characterize pediatric patients with AHRF and hemodynamic instability supported by ECMO and identify prognostic risk factors. The study was approved by the Institutional Ethics Committee of the Children’s Hospital of Fudan University.

### Patient population

We enrolled children (aged 28 days–18 years) with AHRF and concurrent hemodynamic instability requiring ECMO support between January 2015 and December 2021. Exclusion criteria included chronic respiratory disease or incomplete medical/laboratory records. AHRF was defined according to standard criteria: acute onset, bilateral infiltrates on chest radiography, an oxygenation index (OI) ≥16, and respiratory failure not fully explained by cardiac failure or fluid overload (10, 11). ECMO was initiated for progressive failure despite optimized conventional therapies (12). Hemodynamic instability was defined as cardiac arrest or the presence of at least two of the following: heart rate >2 SD above age-normal; systolic blood pressure <2 SD below age-normal; Vasoactive-Inotropic Score (VIS) ≥5; or serum lactate ≥3 mmol/L (13).

### ECMO management

Management followed Extracorporeal Life Support Organization (ELSO) guidelines. Our center performs >20 ECMO cases annually. We utilized centrifugal pumps and Medtronic (Minneapolis, MN) membranes and circuits. All patients received VA-ECMO and no patients were initially placed on VV-ECMO and subsequently converted to VA-ECMO. Cardiac function was evaluated via echocardiography prior to cannulation of the internal jugular vein and carotid artery.

Following stabilization on ECMO, ultra-protective ventilation was implemented: peak inspiratory pressure (PIP) 20–25 cmH_2_O, positive end-expiratory pressure (PEEP) ≥10 cmH_2_O, and tidal volume (VT) 4–6 mL/kg predicted body weight. Sweep gas and blood flow were adjusted to maintain PaCO_2_ between 35 and 45 mmHg. Initial blood flow rates were set at 80–120 mL/kg/min. Tissue perfusion was monitored via mixed venous oxygen saturation (SvO_2_), lactate levels, urine output, and peripheral perfusion. Anticoagulation was maintained with continuous heparin infusion, targeting an activated clotting time (ACT) of 180–220 s and an activated partial thromboplastin time (APTT) <2× the upper limit of normal. Continuous renal replacement therapy (CRRT) was integrated into the circuit for acute kidney injury or fluid management.

### Definitions

The primary outcome was survival to 28 days post-admission. Successful weaning was defined as survival for >48 h post-decannulation without further extracorporeal support (14, 15). Complications were categorized as: bleeding (including intracranial, respiratory, gastrointestinal, or surgical site hemorrhage), thrombosis (clots within the circuit or patient), and mechanical failure (e.g., cannula dislodgement).

### Data collection

We extracted demographics, comorbidities, and pre-ECMO clinical data, including rescue therapies (e.g., CRRT), ventilator settings, and oxygenation indices. Blood gas parameters and VIS were recorded within 6 h prior to ECMO initiation; other laboratory findings were recorded within 24 h.

### Statistical analysis

Analyses were performed using SPSS version 23. Continuous variables are expressed as median (IQR) and compared using the Mann-Whitney U test. Categorical variables are expressed as *n* (%) and compared using Fisher’s exact test. Variables with a *p*-value ≤ 0.1 on univariate analysis [pre-ECMO PaO_2_/FiO_2_ (P/F) ratio, FiO_2_ and PaO_2_] were entered as independent variables into a multivariable binary logistic regression model, with survival set as the dependent variable. The predictive accuracy of the P/F ratio was assessed using the area under the receiver-operating characteristic curve (AUC-ROC), with the optimal threshold determined by the Youden index. Kaplan-Meier survival curves until 28 days after admission were compared with a log-rank test. Statistical significance was set at *p* < 0.05.

## Results

### Patient characteristics

A total of 57 pediatric patients with AHRF and hemodynamic instability were initially enrolled. Following the exclusion of five patients due to incomplete data, 52 patients (91.2%) were included in the final analysis.

Baseline characteristics of survivors and non-survivors are summarized in Table 1. The overall 28-day mortality rate was 53.8%, with no significant difference observed between sexes. The median age was 25 months. Comorbidities were present in 50.0% of the cohort, including hematological malignancies (13.5%), immunodeficiency (13.5%), congenital heart disease (7.7%), and bronchopulmonary dysplasia (13.5%). Viral infection was the leading etiology of respiratory failure. Air leak syndrome occurred in 57.7% of patients, with pneumothorax (34.6%) and pleural effusion (21.2%) being the most frequent complications. The median Pediatric Logistic Organ Dysfunction-2 (PELOD-2) score was 9. Pre-ECMO rescue interventions included CRRT in 21.2% of patients. Notably, 63.5% of the cohort required inter-hospital transport while on ECMO.

**TABLE 1:** General clinical characteristics of extracorporeal life support registry subjects.

Patient characteristics	Total (*n* = 52)	Survivors (*n* = 24)	Non-survivors (*n* = 28)	*P*
Gender: male, *n* (%)	33 (63.5)	18 (75.0)	15 (53.6)	0.106
Age, m median (25%–75%)	25.0 (7.5, 53.0)	20.5 (8.8, 48.5)	33.0 (5.9, 58.7)	0.833
Weight, kg median (25%–75%)	11.0 (7.4, 17.0)	11.0 (7.8, 17.0)	13 (7.1, 17.0)	0.811
Comorbid conditions, *n* (%)	26 (50.0)	10 (41.7)	16 (57.1)	0.266
Hematological malignancies, *n* (%)	7 (13.5)	2 (8.3)	5 (17.9)	0.430
Immunodeficiency, *n* (%)	7 (13.5)	3 (12.5)	4 (14.3)	1.000*
Congenital heart disease, *n* (%)	4 (7.7)	2 (8.3)	2 (7.1)	1.000*
Bronchopulmonary dysplasia, *n* (%)	7 (13.5)	3 (12.5)	4 (14.3)	1.000*
Pathogens				
Bacterial pneumonia, *n* (%)	13 (25.0)	5 (20.8)	8 (28.6)	0.521
Viral pneumonia, *n* (%)	22 (42.3)	10 (41.7)	12 (42.9)	0.931
Miscellaneous, *n* (%)	14 (26.9)	7 (29.2)	7 (25.0)	0.736
Air leak, *n* (%)	30 (57.7)	13 (54.2)	17 (60.7)	0.634
Pleural effusion, *n* (%)	11 (21.2)	4 (16.7)	7 (25.0)	0.463
Pneumothorax, *n* (%)	18 (34.6)	8 (33.3)	10 (35.7)	0.857
Mediastinal emphysema, *n* (%)	6 (11.5)	4 (16.7)	2 (7.1)	0.397*
Subcutaneous emphysema, *n* (%)	3 (5.8)	2 (8.3)	1 (3.6)	0.590*
Vasoactive-inotropic score, median (25%–75%)	8.7 (0.0, 56.8)	8.75 (0.0, 61.2)	7.5 (0.0, 53.7)	0.751
PELOD-2, median (25%–75%)	9.0 (7.0, 13.0)	9.0 (7.0, 12.7)	9.0 (7.2, 13.0)	0.592
Pre-ECMO CRRT, *n* (%)	11 (21.2)	3 (12.5)	8 (28.6)	0.157
Pre-ECMO CPR, *n* (%)	8 (15.4)	4 (16.7)	4 (14.3)	1.000*
Pre-ECMO transportation, *n* (%)	33 (63.5)	15 (62.5)	18 (64.3)	0.894

Data are expressed as median (25%–75% interquartile range) or number (percentage).

*Fisher exact test.

As shown in Tables 1, 2, the pediatric cohort demonstrated high severity of illness with hemodynamic instability, characterized by a median pre-ECMO VIS of 8.7 and a median lactate level of 1.1 mmol/L. There were 15.4% of patients had received cardiopulmonary resuscitation (CPR) before ECMO.

**TABLE 2:** Variables of lung function supported by ventilation pre and post ECMO.

Lung function variables	Total (*n* = 52)	Survivors (*n* = 24)	Non-survivors (*n* = 28)	*P*
Pre-ECMO				
PaO_2_, mmHg, median (25%–75%)	60.7 (50.3, 86.3)	64.4 (50.3, 117.2)	60.5 (49.8, 72.7)	0.011
PaCO_2_, mmHg, median (25%–75%)	58.3 (44.8, 86.3)	58.5 (44.8, 73.9)	58.2 (44.6, 72.6)	0.873
SPO_2_, median (25%–75%)	90.0 (82.3, 94.5)	89.5 (82.1, 98.6)	90.8 (82.1, 93.8)	0.139
Lactic acid, mmol/L, median (25%–75%)	1.1 (0.6, 2.5)	0.8 (0.4, 2.2)	1.9 (0.6, 5.4)	0.120
PH, median (25%–75%)	7.3 (7.2, 7.4)	7.3 (7.2, 7.3)	7.3 (7.2, 7.4)	0.213
OI, median (25%–75%)	36.1 (26.8, 43.1)	32.4 (19.1, 40.3)	36.8 (27.9, 43.5)	0.212
PaO_2_/ FiO_2_, median (25%–75%)	68.0 (54.1, 94.4)	75.9 (54.1, 139.0)	61.8 (50.6, 78.0)	0.007
Mean airway pressure, cmH_2_O, median (25%–75%)	23.0 (19.0, 26.3)	23.0 (19.7, 30.2)	22.0 (19.0, 26.0)	0.399
Positive end-expiratory pressure, cmH_2_O, median (25%–75%)	10.0 (8.0, 14.0)	10.0 (7.0, 12.0)	12.0 (8.0, 15.0)	0.223
FiO_2_, %, median (25%–75%)	100.0 (90.0, 100.0)	90.0 (85.0, 100.0)	100.0 (91.2, 100.0)	0.067
Ventilator days, d, median (25–75%)	4.0 (1.5, 9.0)	3.0 (1.5, 5.0)	5.0 (1.3, 11.2)	0.214
Other rescue therapy, *n* (%)	28 (53.8)	10 (41.7)	18 (64.3)	0.102
High-frequency ventilation, *n* (%)	6 (11.5)	3 (12.5)	3 (10.7)	1.000*
Inhaled NO, *n* (%)	6 (11.5)	1 (4.2)	5 (17.9)	0.199*
Exogenous surfactant, *n* (%)	9 (17.3)	3 (12.5)	6 (21.4)	0.480*
Prone positioning, *n* (%)	20 (38.5)	8 (33.3)	12 (42.9)	0.482
Lung recruitment maneuvers, *n* (%)	6 (11.5)	1 (4.2)	5 (17.9)	0.199*
Post- ECMO				
PaO_2_, mmHg, median (25%–75%)	102.7 (63.5, 251.2)	91.8 (61.6, 270.0)	114.5 (67.8, 251.2)	0.666
PaCO_2_, mmHg, median (25%–75%)	38.5 (32.4, 43.2)	34.1 (29.5, 45.4)	40.3 (36.0, 43.2)	0.135
SPO_2_, median (25%–75%)	96.5 (95.0, 98.0)	97.0 (95.0, 98.0)	95.5 (94.2, 98.0)	0.200
Lactic acid, mmol/L, median (25%–75%)	1.3 (0.8, 2.6)	1.0 (0.7, 2.5)	1.8 (1.0, 3.2)	0.163
PH, median (25%–75%)	7.5 (7.4, 7.5)	7.5 (7.4, 7.6)	7.4 (7.4, 7.5)	0.215
Mean airway pressure, cmH_2_O, median (25%–75%)	15.0 (13.0, 19.0)	15.5 (12.0, 18.7)	15.0 (13.0, 19.0)	0.712
Positive end-expiratory pressure, cmH_2_O, median (25%–75%)	10.0 (7.3,12.0)	9.0 (6.0, 10.0)	10.0 (8.0, 12.0)	0.143
FiO_2_, %, median (25%–75%)	45.0 (40.0, 60.0)	45.0 (40.0, 50.0)	45.0 (40.0, 60.0)	0.357

Data are expressed as median (25%–75% interquartile range) or number (percentage). ECMO, extracorporeal membrane oxygenation; OI, oxygenation index; CRRT, continuous renal replacement therapy; CPR, cardiopulmonary resuscitation.

*Fisher exact test.

### Respiratory function and ventilator support

All patients received mechanical ventilation prior to ECMO. Table 2 details respiratory parameters before and during ECMO support. Survivors had significantly higher pre-ECMO PaO_2_ levels compared to non-survivors (median [IQR]: 64.4 [50.3–117.2] vs. 60.5 [49.8–72.7] mmHg; *p* = 0.011). Although lactate levels were lower in survivors (0.8 [0.4–2.2] vs. 1.9 [0.6–5.4] mmol/L), this trend did not reach statistical significance. Respiratory acidosis and CO_2_ retention were prevalent across the cohort (median pH 7.3 [7.2–7.4]). While the OI was higher in non-survivors (36.8 [27.9–43.5] vs. 32.4 [19.1–40.3]), the difference was not significant (*p* = 0.212). However, the P/F ratio was significantly higher in survivors (75.9 [54.1–139.0] vs. 61.8 [50.6–78.0]; *p* = 0.007).

Regarding pre-ECMO mechanical ventilation, a lung-protective ventilation strategy was universally applied to the pediatric cohort. The median pre-ECMO PEEP was 10 cmH_2_O (IQR, 8.0–14.0 cmH_2_O), and the median Mean Airway Pressure (MAP) was 23 cmH_2_O, (IQR, 19.0–26.3 cmH_2_O) (Table 2). Rescue therapies prior to ECMO included prone positioning (38.5%), high-frequency oscillatory ventilation (11.5%), inhaled nitric oxide (11.5%), and surfactant administration (17.3%). By 24 h post-ECMO initiation, median PaO_2_ increased to 102.7 mmHg (63.5–251.2 mmHg), while mean airway pressure decreased from a pre-ECMO median of 23–15 cmH_2_O.

### ECMO implementation and complications

All patients were supported via VA-ECMO utilizing internal jugular and carotid artery cannulation. The median duration of ECMO support was 208 h, with no significant difference between survivors and non-survivors (*p* = 0.949). The median blood flow rate was 80 mL/kg/min. Membrane oxygenator exchange was required in 32.7% of cases. Complications occurred in 57.7% of patients, including hemorrhage (44.2%), thrombosis (25.0%), and mechanical issues (3.8%). As demonstrated in Table 3, neither hemorrhage (*p* = 0.143) nor thrombosis (*p* = 0.521) was significantly associated with mortality in this pediatric cohort.

**TABLE 3:** ECMO initiation and related complications.

ECMO variables	Total (*n* = 52)	Survivors (*n* = 24)	Non-survivors (*n* = 28)	*P*
ECMO time, h, median (25%–75%)	208.0 (132.2, 371.0)	208.0 (157.0, 256.6)	211.0 (95.8, 453.3)	0.949
ECMO blood flow, ml/kg/h, median (25%–75%)	80.0 (58.8, 93.4)	78.8 (63.1, 93.1)	80.0 (57.2, 97.3)	0.756
Times of membrane oxygenators change	–	–	–	0.052
0, *n* (%)	35 (67.3)	16 (66.7)	19 (67.9)	–
1, *n* (%)	13 (25.0)	7 (29.2)	6 (21.4)	–
2, *n* (%)	2 (3.8)	0 (0)	2 (7.1)	–
3, *n* (%)	2 (3.8)	1 (4.2)	1 (3.6)	–
Complications, *n* (%)	30 (57.7)	11 (45.8)	19 (67.9)	0.109
Bleeding, *n* (%)	23 (44.2)	8 (33.3)	15 (53.6)	0.143
Thrombosis, *n* (%)	13 (25.0)	7 (29.2)	6 (21.4)	0.521
Mechanical complications, *n* (%)	2 (3.8)	1 (4.2)	1 (3.6)	1.000*

Data are expressed as median (25%–75% interquartile range) or number (percentage). ECMO, extracorporeal membrane oxygenation.

*Fisher exact test.

### Clinical outcomes

In total, 24 patients (46.2%) survived to the primary endpoint (Table 4). Successful weaning was achieved in 34 patients and was strongly associated with survival (*p* < 0.001). While survivors tended to have more ventilator days (26 [17.5–41.2] vs. 15.5 [9.0–39.0]; *p* = 0.102), the length of ICU stay was significantly longer for survivors (43.0 [25.5–61.7] vs. 15.0 [7.0–32.5] days; *p* < 0.001).

**TABLE 4:** Outcome of patients.

Outcome variables	Total (*n* = 52)	Survivors (*n* = 24)	Non-survivors (*n* = 28)	*P*
Successful weaning, *n* (%)	34 (65.4)	23 (95.8)	11 (39.3)	<0.001
Ventilator days, d, median (25%–75%)	23.0 (9.2, 39.7)	26.0 (17.5, 41.2)	15.5 (9.0, 39.0)	0.102
ICU length of stay, d, median (25%–75%)	27.5 (9.0, 52.0)	43.0 (25.5, 61.7)	15.0 (7.0, 32.5)	<0.001

Data are expressed as median (25%–75% interquartile range) or number (percentage).

### Multivariable analysis

**FIGURE 1:**
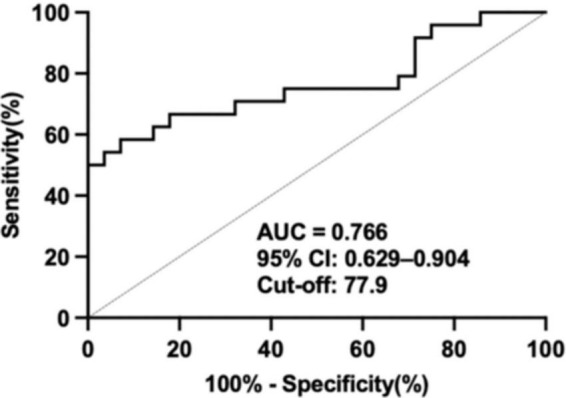
Receiver operating characteristic **(ROC)** curve analysis of pre-ECMO P/F ratio as a predictor of mortality. The area under the curve **(AUC)** is 0.766 (95% CI: 0.629’s of pre- = 0.007). At the optimal cut-off value of 77.9, the sensitivity and specificity for predicting 28-day mortality were 58.3% and 92.9%, respectively. The diagonal dashed line represents the reference level (AUC = 0.5).

**FIGURE 2:**
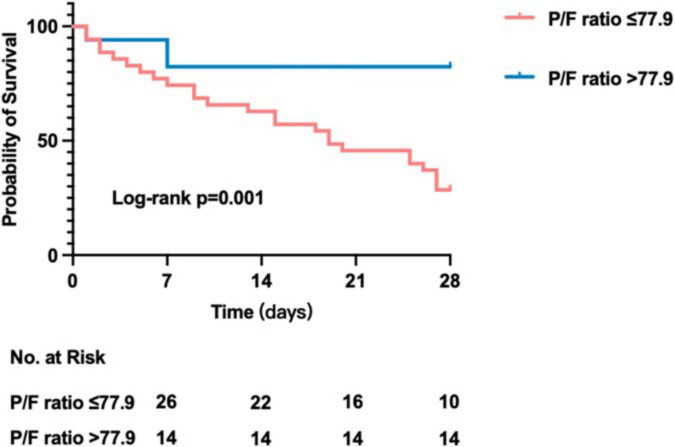
Kaplan-Meier survival curves stratified by the pre-ECMO P/F ratio threshold. The 28-day cumulative survival rate was significantly lower in patients with a pre-ECMO P/F ratio ≤77.9 (red line, *n* = 35) compared to those with a pre-ECMO P/F ratio >77.9 (blue line, *n* = 17). Statistical significance was evaluated using the log-rank test (*p* = 0.001). The numbers of patients at risk at each time interval (0, 7, 14, 21, and 28 days) are presented beneath the grid.

In multivariate analysis, P/F ratio was significantly associated with survival (odds ratio 1.047, 95% CI 1.015–1.081, *p* = 0.004) (Table 5). ROC analysis identified the P/F ratio as a predictor of survival, with an AUC of 0.766 (95% CI: 0.629–0.904) (Figure 1). The optimal P/F ratio cut-off for predicting mortality was 77.9 (sensitivity: 58.3%; specificity: 92.9%; the positive predictive value: 82.4%; the negative predictive value: 71.4%). The internal validation yielded consistent results with the primary analysis (Supplementary Table 1). The hazard ratio for death within 28 days after admission in the P/F ratio ≤77.9 group, as compared with the P/F ratio >77.9 group, was 5.459 (95% CI, 2.560 to 11.64; *p* = 0.001) (Figure 2).

**TABLE 5:** Univariate and multivariable logistic regression analysis of factors associated with survival.

Variables in the model	OR	Adjusted OR (95% CI)	*P*
Pre-ECMO FiO_2_	1.095	(0.871–1.375)	0.437
Pre-ECMO P/F ratio	1.047	(1.015–1.081)	0.004
Pre-ECMO PaO_2_	1.184	(0.895–1.566)	0.377

ECMO, extracorporeal membrane oxygenation; P/F ratio, PaO_2_/FiO_2_ ratio; OR, odds ratio.

## Discussion

Acute hypoxemic respiratory failure remains a formidable challenge in pediatric critical care. Although AHRF affects only approximately 3% of PICU admissions globally and exhibits declining mortality rates (16–18), optimizing rescue strategies for refractory cases remains a critical clinical priority. In our cohort, viral pneumonia (40%), including H1N1 influenza, constituted the primary etiology. Previous literature indicateed that patients with H1N1-related respiratory failure frequently achieve favorable outcomes under ECMO support (19). However, pediatric evidence regarding ECMO efficacy remains equivocal: while certain studies demonstrate a clear survival benefit (20), others reported no significant advantage over conventional mechanical ventilation (21). These conflicting data largely stem from the non-randomized design of pediatric ECMO research and the inherent difficulty in controlling for myriad confounding variables.

In pediatric ARDS, clinical consensus typically positions VV ECMO as the first-line support. However, patients presenting with concomitant hemodynamic instability or cardiogenic shock require VA ECMO to provide comprehensive, biventricular and pulmonary support. Our study yielded an overall mortality rate of 53.8%. This outcome aligns closely with the 56% mortality reported in similar high-acuity cohorts, yet stands notably higher than the mortality typically associated with isolated respiratory failure managed via VV-ECMO (22). To our knowledge, this work represented the largest single-center study in China to evaluate the association between clinical parameters and outcomes in pediatric patients requiring VA-ECMO for concurrent refractory hypoxemia and hemodynamic compromise.

The optimal timing for pediatric ECMO initiation remains highly debated. Although international trials have failed to establish a definitive benefit for “early” ECMO deployment in general ARDS (23), our findings suggested that delayed initiation may compromise survival in patients with multi-organ involvement. Specifically, higher pre-ECMO PaO_2_ levels and P/F ratios significantly correlated with improved survival. Our ROC analysis identified a P/F ratio threshold of 77.9 as a highly specific (92.9%) predictor of survival. While the Second International Guidelines for the Diagnosis and Management of Pediatric Acute Respiratory Distress Syndrome emphasizes both the P/F ratio and OI for grading ARDS severity (11), adult guidelines extrapolating from the EOLIA trial eligibility criteria—recommend ECMO referral for severe ARDS defined by a P/F ratio <50 for >3 h, or <80 for >6 h (24). Our data suggested that within the specific context of VA-ECMO, the P/F ratio served as a more sensitive prognostic marker than the OI. This discrepancy likely arises because the OI depends heavily on the mean airway pressure, a parameter that varies substantially based on individual institutional ventilation practices prior to cannulation.

Previous studies have identified prolonged mechanical ventilation prior to ECMO — particularly exceeding 14 days—as a robust predictor of mortality (25, 26). In our cohort, the median duration of pre-ECMO ventilation was 4 days, with no significant difference observed between survivors and non-survivors. This finding indicated that the severity of the initial physiological insult (as reflected by the P/F ratio), rather than the duration of preceding ventilation, exerted a more decisive impact on clinical outcomes in this specific population.

Contrary to historical reports, age, comorbidities, and baseline lactate levels did not emerge as independent predictors of mortality in our study (27–30). This divergence may stem from our highly selective VA-ECMO cohort, where severe hemodynamic compromise served as a primary inclusion criterion, potentially overshadowing the prognostic impact of baseline comorbidities. Furthermore, although complications such as bleeding (44.2%) and thrombosis (25.0%) occurred frequently, they did not directly drive mortality. This highlight underlines the critical role of standardized anticoagulation and transfusion protocols in mitigating these high-stakes risks (31–35).

## Limitations

Our study has several limitations. Firstly, we acknowledge that sample size was not predetermined given the retrospective design of the study. We performed a *post hoc* power analysis. Given our sample size of 52 patients, an observed survival difference of 82.4% in high P/F ratio group and 28.3% in low P/F ratio groups, and α = 0.05, the achieved power is 0.96. Secondly, this study was constrained by its relatively small sample size, which inherently carries a potential risk of model overfitting. However, this risk is considered manageable, as our internal validation via 1,000 bootstrap replicates yielded risk estimates highly consistent with the primary analysis, thereby reinforcing the stability of our findings. Thirdly, the lack of a control group managed without ECMO limits our ability to make definitive statements regarding the comparative efficacy of VA-ECMO. Additionally, due to the emergency and retrospective nature of our pediatric cohort, advanced respiratory mechanics such as driving pressure and static compliance were unavailable. Caution should be exercised when generalizing our findings to broader clinical settings or other pediatric intensive care populations. Further multicenter investigations with larger sample sizes are essential to corroborate our conclusions.

## Conclusion

Lower pre-ECMO P/F ratio was associated with a poor prognosis in pediatric patients suffering from severe AHRF complicated by hemodynamic instability managed with VA-ECMO. Utilizing this metric may enhance early risk stratification and optimize candidate selection, though future multicenter studies are warranted.

## Data Availability

The raw data supporting the conclusions of this article will be made available by the authors, without undue reservation.
