# Supplementary material for: Evaluation of extracorporeal membrane oxygenation in children with acute hypoxemic respiratory failure and hemodynamic instability in China: a 7-year single-center retrospective study
Source: Front Med (Lausanne). 2026 Jun 24;13:1867681. doi: 10.3389/fmed.2026.1867681 (PMC13341832; doi:10.3389/fmed.2026.1867681)
Supplement: Supplementary file 1 [file Table_1.docx]

Table S1 Bootstrap-adjusted risk estimates for the ROC-derived pre-ECMO P/F ratio

|  | Value | 95% Confidence Interval |
| --- | --- | --- |
| Odds ratio for pre-ECMO P/F ratio | 1.090 | (1.020-1.372) |

P/F ratio, PaO_2_/ FiO_2_ ratio; ECMO, extracorporeal membrane oxygenation.
